# Food Security in the Context of Paternal Incarceration: Family Impact Perspectives

**DOI:** 10.3390/ijerph16050776

**Published:** 2019-03-04

**Authors:** Karen M. Davison, Carla D’Andreamatteo, Sabina Markham, Clifford Holloway, Gillian Marshall, Victoria L. Smye

**Affiliations:** 1 School of Nursing, University of British Columbia, 2211 Wesbrook Mall, Vancouver, BC V6T 2B5, Canada; Sabina.Markham@macl.bc.ca (S.M.); vsmye@uwo.ca (V.L.S.); 2 Fulbright Canada Visiting Research Chair, College of Social Sciences, University of Hawaii at Mānoa, 2500 Campus Road, Hawaii Hall 310, Honolulu, HI 96822, USA; 3 Health Science Program, Department of Biology, Kwantlen Polytechnic University, 12666 72nd Avenue, Surrey, BC V3W 2M8, Canada; 4 Food and Nutritional Sciences, University of Manitoba, 209 Human Ecology Building, Winnipeg, MB R3T 2N2, Canada; carla@thefoodlady.ca; 5 University of Ontario Institute of Technology, Health Science, 2000 Simcoe Street North, Oshawa, ON L1G 0C5, Canada; cliffholloway@hotmail.com; 6 Department of Social Work, University of Washington, 1900 Commerce Street, Tacoma, WA 98402, USA; geegee@uw.edu; 7 Arthur Labatt Family School of Nursing, Western University, Room 3306, FIMS & Nursing Building, London, ON N6A 3K7, Canada

**Keywords:** food insecurity, incarceration, ethnography, family, community, intersectionality

## Abstract

Although research about the unintended consequences of paternal incarceration for family well-being has grown in recent years, there has been minimal exploration of food insecurity. Using qualitative methods, we aimed to understand the relationships between paternal incarceration and family food insecurity in Canada. An ethnographic study (24 months) was conducted that included naturalistic observation and in-depth interviews with formerly incarcerated fathers, their partners, and societal reintegration-focused stakeholders (*n* = 63). Interpretive thematic analysis based on family impact and intersectional theories, indicated that family food insecurity was elucidated by pre-incarceration, economic, social, health, and relationship factors; stigma and social/structural constraints; and intersections among individual, correctional system, community, and macro-level (i.e., economic, social, policy, physical contexts) factors. Participatory approaches and collaborative action among diverse stakeholders that include practitioners, policy makers, researchers, as well as health, social, and criminal justice agencies can guide best practices in creating supportive food environments for families impacted by adversities of incarceration. In particular, interventions aimed at prescriptive ethics, social justice, and meaningful rehabilitation show promise at mitigating the collateral consequences of incarceration-related food insecurity.

## 1. Introduction

In Canada, a substantial proportion of males who serve time in prison are fathers [1,2], and this exposes numerous families to the collateral consequences of incarceration. Paternal incarceration contributes to economic, social, and health inequalities [3,4] which are associated with physical and mental health [5,6] as well as, food insecurity [7]. However, there is limited understanding about family food insecurity in this context which impedes progress in programs and policies.

Food insecurity—the tenuous access to sufficient, safe, and nutritious food to meet dietary needs—is among one of the inter-related economic, social, relational and familial impacts that occurs with paternal incarceration [8]. Incarceration contributes to economic instability, a known correlate of food insecurity [9], and to human and social capital deficits [3] which can incapacitate a family’s ability to consistently access appropriate food. Collateral consequences of paternal incarceration that can impact food security [10] include lower contribution of shared earnings with an intimate partner [11], destabilized family relationships, impaired parenting behaviors [12,13,14], increased mental health and behavioral problems [15], health care access challenges [14], barriers to reintegration [2,16,17] and the inter-generational transmission of inequality [2].

When a father is incarcerated, the child’s mother tends to be the primary caregiver. While data on the global rates of paternal incarceration is lacking, it is estimated that about 5% of Canadian children are affected by paternal incarceration [2]. Although research about the repercussions of fathers being incarcerated has burgeoned in recent years, there has been limited exploration regarding the contributions of food insecurity and its familial impacts. For example, Turney [18] analyzed data from the Fragile Families and Child Well-Being Study using propensity score matching models and reported that paternal incarceration in the previous 2 years doubled the likelihood the father’s children (at 5 years of age) reported food insecurity. Two studies [19,20], that examined incarceration-related food insecurity and food-diet experiences during incarceration discussed links between maternal incarceration and their child’s later-life experiences with food insecurity and highlighted how diet modifications while incarcerated can be a stressor. While these investigations have provided important insights, most have been conducted in the U.S. and may not necessarily detect nuanced, historical, cultural, and dynamic experiences specific to Canada. To contribute to knowledge about paternal incarceration and family food insecurity, a qualitative study was undertaken that focused on fathers who had the experience of incarceration in the Canadian federal correctional system (i.e., serving or had served a custodial sentence of ≥ 2 years). Paternal incarceration was examined because its cumulative risk has been shown to be much greater than that of maternal imprisonment [21,22]. The goals of this investigation were to examine how: (1) a father’s experiences with food while incarcerated may impact on individual and family level food insecurity; (2) paternal incarceration impacts family food insecurity; (3) different factors contribute to family food insecurity in the context of paternal incarceration; and (4) stakeholders who work with individuals affected by paternal incarceration (e.g., from correctional, health, social organizations) perceive and address the issue of food insecurity.

## 2. Methods

### 2.1. Research Setting

In Canada, responsibilities for corrections are shared by federal, provincial, and territorial governments [23]. Correctional Service Canada (CSC) is the federal agency and is headed by the Correctional Service Commissioner who reports to the Minister of Public Safety Canada and is supported by an executive committee of national and regional members. Federal corrections are concerned with offenders who have been sentenced for two years or more. Provincial and territorial corrections are responsible for young and adult offenders who have been sentenced for two years less a day (or less) or who have received community sentences such as fines, community service work, or probation. There are two types of court-imposed federal sentences. The first, a determinate sentence, has a specified completion date, after which CSC no longer has jurisdiction over the offender. The second, an indeterminate sentence, means that CSC has jurisdiction over the offender until the offender passes away. With an indeterminate sentence, the court imposes a minimum amount of time before the offender can apply to the Parole Board of Canada for conditional release. For example, a court-imposed sentence of life with no parole for 25 years would indicate that the offender would be incarcerated for at least 25 years prior to consideration for a potential conditional release to the community, under the supervision of a community parole officer. Once a sentence is imposed, a thorough intake assessment determines the offender’s risk level, needs, and initial security level placement. Maximum security institutions are the most restrictive. They house individuals who pose the greatest risk of escape and danger to society. The inmate’s daily schedule is structured and strict. In medium security institutions, officers are not armed and daily life is similar to that in maximum security facilities. The main functions of minimum security institutions are to foster successful societal reintegration. These facilities are usually organized as small communities where inmates reside in living units (houses) in small groups. There are no barbed wired fences or armed officers, as inmates in these institutions are deemed very low risk. Inmates can organize their schedule according to the activities they are required to participate in (e.g., work programs) and often are responsible for their own meals. While no recent data is available about fathers who are incarcerated, it is estimated that males account for 83% of adult admissions to correctional services in the provinces and territories. For federal corrections, males account for 92% of custody and community admissions [24].

The study took place in the Fraser Valley of British Columbia where there are six federal correctional institutions for male offenders. The CSC-managed facilities in the region include all security levels (minimum to maximum; some are multi-level) and all are located within a 60 km radius. The investigation was a collaboration of the University of British Columbia’s School of Nursing and two community agencies, Long-term Inmates Now in Community (L.I.N.C.) and Hope Central. L.I.N.C. is operated by ex-offenders and provides services (including food provision) to people impacted by the criminal justice system (e.g., offenders, parolees, and ex-offenders, individuals who support them, victims). Members of L.I.N.C. come from a wide range of social and economic backgrounds. Hope Central is a centre operated by a community church that provides drop-ins and programming that focuses on relief (e.g., provision of basic supplies), rehabilitation, and life skills development to marginalized individuals including those who have been incarcerated.

### 2.2. Study Population

For this study the population of interest were families where: (1) at the time of study the father was currently or formerly incarcerated in a Canadian federal correctional facility; (2) at the time of the arrest the father either had custodial (i.e., living with their children) or non-custodial (i.e., had children with whom they were not living with) status; and (3) access to food was indicated as an issue during incarceration and/or reintegration by the participant and/or community partner(s) working with that participant.

Candidates for stakeholder interviews were selected based on criteria that included: (1) providing CSC and/or affiliated services; (2) having expertise in working with individuals that have lived experience of being incarcerated in a CSC facility and/or their families; and/or (3) providing food related programs or services that include individuals that are currently or formerly incarcerated and/or their families. Although previous work has suggested that stakeholders’ insights can diverge from those with the lived experience food insecurity [25], including their perspectives was deemed to be important in ascertaining how they may be barriers in the perpetuation of inequality to food access.

### 2.3. Data Collection

#### 2.3.1. Naturalistic Observation and Ethnography

More than 570 h of naturalistic observation data was collected by two researchers over 16 months during drop-ins of Hope Central (62 drop-ins attended with an average number of 38 participants per drop-in), community garden program organized by L.I.N.C. that engaged current and former incarcerated individuals (31 visits with an average of 6 participants), and weekly support groups of L.I.N.C (43 visits with an average of 15 participants). In addition, visits to CSC facilities and other community-based programs were done to better understand the range of services that address food insecurity. Naturalistic observations were recorded as brief aides-memoire [26] with extensive field notes documented after site visits. The notes were organized into four categories: (1) details of visit; (2) sensory impressions and personal responses; (3) specific conversations and insider language; and (4) questions for future investigation. The notes were open coded to keep the inquiry broad and develop a list of exhaustive themes. Examples of preliminary codes included “control of dignified food access”, “expectation of community services”, and “need for advocacy”. Over time, the codes were refined to generate more meaningful categories.

An ethnographic approach was used to learn about experiences of paternal incarceration and its culture, values, beliefs, behaviors, and practices [27]. The researchers spent time in the different settings to watch, listen, and engage with the individuals before theorizing the observed phenomena [26,28]. In particular, the researchers considered power relations, values [29], and influences on food insecurity as a means to support changes [30].

#### 2.3.2. In-Depth Individual and Focus Group Interviews

Purposive and theoretical sampling was used to gather in-depth interview data on practices and perceptions related to food insecurity and paternal incarceration from a diversity of participants and stakeholders (Table 1). One set of data was collected from formerly incarcerated fathers and their family members. These interviews focused on gathering information about the father’s experiences of accessing food during incarceration and reintegration and the family’s experiences with accessing food in their community. Another set of data was collected from various stakeholders that focused on gathering information about factors related to appropriate food access during incarceration and reintegration and perspectives about how to address food insecurity in the context of paternal incarceration. Interviews took place at offices of the community agencies and in participants’ homes and ranged from 40 to 125 min (average 85 min). With permission, interviews were audiotaped and professionally transcribed.

Focus groups were also conducted that included family members with the lived experience of food insecurity related to paternal incarceration and stakeholders. The purpose of the focus groups was to explore disparate views, enable point-counterpoint discussion and resolution, and to extract latent issues. The final sample size was determined as the point in data collection where redundancy in themes occurred.

The individual and focus group interview guides are located in the Supplementary file.

#### 2.3.3. Ethics Statement

Participation in the interviews was entirely voluntary and had no effect on the receipt of any services. Written informed consent was obtained prior to each individual and focus group interview. Reimbursement for expenses related to interview attendance (e.g., parking, transportation) was provided. The study was approved by the University of British Columbia’s Behavioural Research Ethics Board and Memorandums of Understanding were signed between the community agencies and the research team (UBC BREB NUMBER: H 12-02095).

### 2.4. Data Analysis

Data was analyzed by six research team members using interpretative thematic analysis. Initially, transcripts were organized and coded as relevant passages of text. As data collection progressed, field and interview notes were read repeatedly to identify patterns and linkages to theory [31]. Transcript codes were compared to identify similarities and differences through discussions among team members to refine categories and themes. Using NVivo Version 10 [32], exemplars of coded text were extracted and compared within and across transcripts. Interpretations were reviewed by team members, stakeholders, and individuals with lived experience of incarceration to check for validity.

To relate participants’ experiences with incarceration and food insecurity, the findings were interrogated based on intersectional [33,34] and family impact [35] theories. Like other scholars [36,37,38], we recognize that disparities arising from factors such as biological sex differences, gendered experience, ethnicity, and class affect food insecurity in ways that are independent and that intersect and compound one another. In addition, we acknowledge that this issue needs to consider how current policies and programs are either barriers or facilitators of family empowerment and stability [35]. In the final analysis, coded narratives were contextualized into a conceptual framework to better understand the multi-level individual, social, and environmental correlates influencing food insecurity and to help provide a clearer picture of the higher impact, upstream forces that may be used to address food insecurity in the context of paternal incarceration [39].

For conceptual clarity, the definition of food insecurity adopted for this study was “whenever the availability of nutritionally adequate and safe foods or the ability to acquire acceptable foods in socially acceptable ways is limited or uncertain” [40]. This definition, supported by ethnographic research [41,42], means that food insecurity is experienced when there is uncertainty about future food availability and access, insufficiency in the amount and kind of food required for a healthy lifestyle, and/or the need to use socially unacceptable ways to acquire food. The term family food insecurity used in this study refers to food intake that is sufficient for family members to lead ‘active, healthy lives’ [43] and accounts for specific cultural relationships with foods [44].

Although the data analysis was situated within the context of systemic inequities that impact individual and family levels of food security [45] which can inform social policy, where applicable the role of broader community levels of food insecurity was questioned. Community food insecurity occurs “when dominant food systems fall short in terms of social, economic and environmental sustainability” [46] and thus provides a foundation that can inform food policy.

Several quality control measures were integrated in the analysis. As part of the study, two peer researchers (formerly incarcerated father and wife of an incarcerated father) were recruited to be part of a research team to help gather and interpret individual and focus group data. Triangulation of data from the fathers and family members, diverse stakeholders, and observational data also contributed to the rigor and trustworthiness of the analysis. Results are reported for areas where most (e.g., >75% of participants) shared similar views.

## 3. Results

The fathers (*n* = 13 who did individual or focus group interviews) who participated in the study had served either determinate or indeterminate prison sentences ranging from two to more than 20 years in different federal institutions (Alberta, British Columbia, Manitoba, Ontario, Nova Scotia). They ranged in ages from 33 to 64 years, had between 1–3 children, and identified themselves as Caucasian, Aboriginal, Asian, or Hispanic. Most (*n* = 10) had at least high school education; three had completed an undergraduate degree. All were custodial fathers prior to incarceration; at the time of the study four participants were no longer with their partners. Some had served more than one prison sentence (federal and/or provincial). Five of the 10 partners of currently or formerly incarcerated individuals who participated were connected with the fathers in the study. At the time of the study all participants were living in British Columbia. The stakeholders (*n* = 40) who participated in individual or focus group interviews were based in different locations across Canada. They included individuals who were employees of CSC (corrections officers, probation officers, food service and programming staff) and employees of agencies that provided integration services (e.g., halfway houses, vocational services, food programs).

### 3.1. Pathways between Paternal Incarceration and Family Food Insecurity

Analysis of the textual data clarified different pathways between paternal incarceration and family food insecurity that included: (1) pre-incarceration experiences/lifestyles and intensified family food insecurity; (2) changes in relationships and health status; and (3) the fathers’ experiences and parental relationship quality.

#### 3.1.1. Pre-Incarceration Experiences and Intensified Family Food Insecurity

Consistent with the literature about paternal incarceration, some participants reported histories of significant economic hardship and most made reference to their experiences of childhood adversity [47]. The described adversities have been reported elsewhere to be associated with food insecurity [19].

*She (mom) made her choices. She had me when she was young. 16. I have no ill will towards her at all..I had no family support…My dad is doing time I think in the States. That is karma. Right? That is what I chalk that up to. Hopefully my kids won’t turn out the same.*

(Father; F#3)

*He came from a hard life and his family were involved with criminal activities in order to make ends meet. My son knows this and about his father being in jail. Even though dad is out we have problems….it worries me that [son’s name] will also think that crime will be an answer for money problems.*

(Wife of formerly incarcerated father; WFI#1)

In addition to the economic hardships, the fathers and various stakeholders also described new or intensified incarceration-related experiences of food insecurity and how this perpetuated intergenerational effects of disadvantage and marginalization. For example, one of the Aboriginal spouses described how incarceration impacted their access to traditional foods.

*We were generally able to eat whatever we wanted as a family but after (father’s name) went to jail, we had to plan meals more carefully and watch costs (before incarceration). We would eat fish which (father’s name) would catch. While he was is jail we ate no fish..(father’s name) wasn’t here to catch it or show the kids how to do this.*

(WFI #4)

In other instances, spouses discussed how their mental health struggles worsened due to financial stresses which imposed constraints on eating. They described how these constraints led to patterns of dietary restriction followed by overconsumption that became cyclical and similar to binge-eating. Other research has reported significant associations between mental ill health and disordered eating [48] and respondent’s descriptions supported the “food stamp cycle” hypothesis which suggests that binge-eating behaviors occur when access to food increases (e.g., availability of food stamps) which is then followed by periods of involuntary restriction when food resources run low [49].

*It’s like the welfare Wednesday situation where you binge spend after you get the cheque..you binge eat when you have the money.*

(WFI#3)

In rare instances, a father’s incarceration had little impact on their family’s access to food, which was consistent with other research insights [19]. This was particularly the case if the father did not live with the family or did not substantially contribute to the family’s income prior to their incarceration. Finally, pre-incarceration factors such as education, social support, and prior food skills were discussed as resources that helped families be resilient to incarceration-related food insecurity.

*..They realized you’re very well educated. This is my diploma. They didn’t know what to do with me. What do you do with a highly educated inmate?.. I really don’t need your help..I’m fine.*

(F#5)

#### 3.1.2. Changes in Relationships and Health Status

Prior to incarceration, most fathers contributed economically to family life, were actively involved in parenting their children, and family food insecurity was not perceived to be an issue. Participants identified incarceration-related factors such as trying to maintain housing security, loss of social connections, loss of work, changes in social assistance, accumulation of legal and household debts, and inability to provide financially for their families which impacted on both economic and food security [50]. Feelings of shame, failure, and inadequacy and how these were motivational barriers in seeking healthy foods were often highlighted.

*..if finances are an issue then no doubt food insecurity becomes an issue. So as soon as that’s an issue then there is a whole host of problems… their social support system is a problem…they don’t leave all of a sudden and have a bunch of new friends that are going to allow for a new life.*

(Stakeholder; Reintegration; SR#8)

*..basically what you’re doing is that someone commits a crime in their home community and they get extracted from the community where they live, they get all their ties, their family, their friends, their social status gets severed and they get pulled out and get socialized with a large population of offenders...so they get basically taken from any pro-social healthy environment... for a long stage of time.*

(Stakeholder; Reintegration; SR#3)

*While in prison I had no real sources of income. You can do work while in jail but they pay less than minimum wage. So yeah..I had nothing to contribute to family finances..[Wife’s name] was on her own..making sure the kids had a roof over their head and food on the table.*

(F#1)

Adding to the economic burdens associated with paternal incarceration, the partners of the fathers reported experiences of significant social network disruptions with family, friends, and community that also placed constraints on food access. They described periods of time where foods consumed were insufficient, low quality, or undesirable. They also discussed how they worried about where to get food and felt forced into obtaining foods in socially unacceptable ways or consuming foods which did not meet personal standards of acceptability.

*I can remember once when [husband’s name] was in prison...I was stone broke... I was on E.I...so I had to go to the food bank…so what happens is the stuff I get from the food bank was white sugar, canned fruit...crap that I couldn’t use. One of the things that I’m very conscious about in terms of prison is the lack of choice, the lack of empowerment around defining your own existence and that’s the same around food…*

(WFI#4)

For partners that resided in smaller communities, they noted how they would try to avoid seeking out food from community-based sources such as food banks to avoid being highly visible and stigmatized. Instead, they would try to obtain alternative financial sources, including engaging in illegal activities, in order to obtain food.

*So I started selling contraband smokes just so I could have some money for us to have a decent life...I didn’t like doing this...but your back is up against a wall...what are ya gonna do?*

(WFI#2)

These comments suggest that the relationships and consequences of incarceration-related food insecurity are bi-directional which has been reported elsewhere [5,51]. Food insecurity either worsened or became a new issue families had to face when the father was incarcerated. Furthermore, these challenges forced families to resort to criminal activities as a means to address their food insecurity. Thus, the relationships between food insecurity and incarceration appeared to be shaped by systemic factors that facilitated mutual reinforcement [52].

Several respondents discussed how the pursuit of meeting basic needs and despondency contributed to the neglect of care for both themselves and their families. Further to this, parents described how these obstacles contributed to the deterioration of their family’s health and how the added disadvantage of food insecurity worsened physical, mental, and social well-being [53,54].

*When (partner’s name) was in prison I was overwhelmed...lawyer’s bills, losing friends..I became severely depressed. I didn’t want to cook...so for me and [son’s name] it was a lot of meals from a can...It was not surprising that problems started happening with [son’s name]. He was acting out at school..*

(WFI#5)

These findings are consistent with other evidence that suggests paternal incarceration is associated with poor eating behaviors, academic and socioemotional skills deficits [55,56,57], antisocial and criminal behavior, internalizing symptoms, mental health problems [58,59,60] and drug use [61] among children whose parents have been incarcerated [52,62]. Furthermore, shared biological pathways among these behavioral (e.g., stress response), mental health (e.g., anxiety over having continued access to appropriate foods), and nutrition-related (e.g., inadequate intake of nutrients due to lack of access to health promoting foods) factors could exacerbate their food insecurity [63]. Macro-level drivers such as food-related policies (e.g., agricultural subsidies) and design of food environments (e.g., retail access to healthy foods) can enable and perpetuate a vicious cycle which relegates the family to society’s fringes and reinforce their struggles with food insecurity [64].

In some instances, family members became formally diagnosed with chronic health conditions (e.g., diabetes, dyslipidemia), a common experience related to incarceration [65]. Often these conditions required adhering to therapeutic diets. Participants spoke about how experiences of food insecurity both seemed to lead to the diagnosis and impede their ability to manage their chronic condition.

*(Daughter’s name) got diabetes and had to insulin. I think the bad diet we had to follow led to this…and now..now with the diabetes it makes things harder with diet …getting the right foods needed to keep her (blood) sugar under control is a huge problem..*

(WFI#4)

It is widely known that health conditions, as described by respondents, arise through a complex web of interactions between genetic and environmental factors including food access [66]. This raises fundamental questions about how to shape social and health practices and policies that prevent gene-diet interactions that lead to chronic condition development. Although there is still much to be done to identify these interactions, this work is relevant to preventing and effectively managing chronic conditions and reducing health, social, and correction service-related costs.

#### 3.1.3. The Fathers’ Experiences and Parental Relationship Quality

Participants shared reports of strained family relationships, including challenges with maternal parenting. These issues were heightened by factors such as the geographical proximity of the father to his family and the type and length of prison sentence. For example, a father who was institutionalized in a province where his family was not residing stated:

*..I never saw them…for almost a year. Which is critical for two little boys right? A whole year with no family. Not as hard on me as it was on those two boys. You know “where is daddy?” and they don’t really understand right? It was the distance. The cost. At the time I was in jail…I had no income… and they don’t understand.*

(F#3)

Fathers described how these circumstances lead to few or no visits with their family and the opportunities that could occur with those visits to share experiences and connections around food. Furthermore, circumstances which lead to disruptions in family meals could impact on children’s biopsychosocial well-being [67]. Within the participant narratives, contextual factors such as facility differences which impacted on the father’s experiences and relationships with food were also highlighted.

*When I was in high security access to food was dictated by the cafeteria. Healthwise I wasn’t doing great…very depressed. I had no control over what I could eat. When I moved into prisons where we could make our own food my relationship with food got better. I could choose what I wanted to buy from the prison’s store. I felt less like a degenerate.*

(F#7)

As others have suggested, choices about food in correctional facilities are different than choices about food in the community [68]. Meal choices are planned by facility staff and those who are incarcerated typically do not provide input about foods offered or eating schedules. Similar to other findings [20], respondents discussed how access to appropriate foods would enable them to manage their health and to be physically and mentally prepared for reintegration. In extreme cases, some discussed how the system of food provision in correctional facilities negatively shaped their relationships in food. Examples were described of how limited food access during lock downs and witnessing violent fights over prized food items made them associate food with traumatic experiences. Many also discussed how correctional system fiscal constraints that often led to food budget cuts contributed to taking the pleasure out of eating and losing a sense of control over food choices. As others have noted [69], perceptions about inadequate food within correctional facilities can fuel frustration, humiliation, and deterioration in health status. This, in turn, can increase risk for rule violations, violence [70], and recidivism [71] which impact the management of correctional facilities.

*So they gone regional [centralized food production—to save money. There is fewer staff..(The prisoners) are not happy. It is one of the few things that get them through the day—the food—and now they see that as being taken away from them. This, among other things that happen in prison, beats them down...*

(Stakeholder; Corrections System; SC#14)

Although respondents acknowledged they were low on the social hierarchy, they discussed how being at this tier also had put them in positions of variable access to food while incarcerated and that this had carryover effects during reintegration.

*…in prisons there is a hierarchy and food certainly played into this. At mealtimes certain inmates got better treatment...better food. Certain inmates would be chosen to work in the kitchen and had more access to food.*

(F#9)

In some cases, social locations such as age, ethnicity, and incarceration experiences influenced their positioning within the carceral hierarchy and either facilitated or impeded food access.

*Some were more likely to have less food available to them. Like the younger ones...they don’t get full. Others may have food brought in for them or be given money by family to use for canteen. Food was used as a commodity on the inside. Some would beg for food from others. Others would steal food.*

(F#5)

*..so you get a certain amount of respect for going through the maxes and what not. I couldn’t care a less. I didn’t care about that…And these guys right would like come and sit at the back table (at meals).and ask..Do you need this? I am just like no it is all good. I’ll just sit with the natives.*

(F#3)

While most discussed food insecurity-related challenges, some noted that being in correctional facilities could provide opportunities to positively influence their eating experiences (e.g., learned food skills) and foster food security during reintegration:

*Some of them have been involved in culinary arts programs. Some guys develop niches where they can maybe bake. So in exchange for baking the other guys will get the ingredients. ..Things like this can help them when they get out.*

(Stakeholder; Reintegration; SR#3)

*While he was in there he actually learned how to cook. Even got his FoodSafe.*

(WFI#4)

Many made reference to how knowledge about health, food, and CSC policies helped to mitigate issues related to appropriate food access. One spoke at length about how he studied policies and prisoner’s rights in depth in order to lobby for better foods.

*We knew our rights..we had access to Stockwell Day’s..pathways to safety..it was some big 400 page manuscript he..that Stockwell Day wrote..in 2007. We got freedom of information on it and got a copy of it. It said things like medication and food proportion weren’t suppose to be messed with.*

(F#3)

### 3.2. Intersecting Factors Impacting Family Food Insecurity

Various intersecting factors shaping experiences of food insecurity were apparent in the participants’ narratives. The main subthemes included associated stigmas of incarceration and the social and structural constraints/supports that affected one’s personal sense of control related to food.

#### 3.2.1. Incarceration and Its Associated Stigmas

Stakeholders in particular discussed how various personal factors such as mental health status, race/ethnicity, and age intersected with problems of knowledge, attitudes (prejudice), and behavior (discrimination). Furthermore, these factors created additional barriers to food security during incarceration and reintegration [72,73]:

*…there was this attempt to treat everyone the same and in some instances that just did not work. For example, the older inmates wouldn’t always get the things they needed...those not from Canada...same thing...they didn’t always get their cultural needs addressed. It would depend...If you’re Aboriginal, you may or may not get accommodated more. The thing is, all the staff would know these people’s histories, so in some ways they wouldn’t be given a chance...*

(SR#12)

*My experience was that, not from the inmates themselves, but rather from the people within the food system...not always a lot of...understanding or belief about the impact of food...I don’t feel there was a lot of credibility given to that area of life and potential positive that could come from it…I guess stigma...comes into that. There is the thought that…well you’re in jail so why should you have good stuff...*

(SR#9)

Instances where the stigmatization of incarceration impeded food access were also voiced by family members. For example, invitations to attend food-related social gatherings became less frequent for families when the father was incarcerated. Others described how participating in activities such as community gardens or community kitchens were impacted. For example, one mother described how she no longer went to weekly community kitchens because people would always be asking about her spouse and the reasons why he was incarcerated.

*I thought going to a community kitchen while (spouse’s name) was in jail would help me make friends and bring home healthy food. But going to them became a chore..I thought I would find support there..instead I felt like I didn’t belong because my reasons for needing food didn’t seem legit to others.*

(WFI#3)

#### 3.2.2. Social and Structural Constraints/Supports

Applying an intersectional approach to the analyses advanced understanding of how social and structural factors such as historical contexts and political will contributed to food insecurity. Respondents discussed how incarceration limited upward social mobility by preventing them from developing both human and social capital [52]. For example, one father’s conviction prevented him from returning to his work as an airline pilot. Furthermore, his highly skilled wife with post-secondary education had to leave her job to help raise their young children. After the father was released, he was forced to work in jobs that paid about one-quarter the wages he was accustomed to and the wife did not return to work. In this instance, both parents who were high income earners lost both individual and social capital as a result of the father’s incarceration. Further to this, participants discussed alternative approaches to incarceration that could facilitate the re-establishment of positive social ties after release as well as mitigate the impacts of food insecurity. Echoed in their narratives were descriptions of how various social and structural constraints blocked positive action.

*..on a completely theoretical and idealistic perspective we would do a deinstitutionalization type approach. So rather than institutionalizing our system it would be community based...If they provide a sentence of more than two years..institutionalization is mandatory. Ideally speaking, we would move away from that if rehabilitation is our goal. But we would need a social mandate to do that and I don’t think we’re anywhere close to that in our society and culture...society wants people who commit a crime to be locked away…So politicians..they want to appeal to voters...public safety is a concern and fear... from a reintegration and rehabilitation perspective that is unhealthy.*

(SR#5)

In addition to the losses in human and social capital, structural constraints specific to food environments were discussed as being barriers to food security [74] both in the carceral setting and during societal reintegration. As other research has shown, incarcerated individuals that reintegrate often live in areas with low access to healthy food retailers which can aggravate the inequity of food insecurity [75].

*..our food system…is an essential and critical part of our environment. And that environment that we create has major impacts on both our current…choices as well as our long term cause and effect stuff. So outside of the prison system, for example, the choices we have at...grocery stores has a major impact…in the environment that we create in the prison or outside the prison or the social circles and networks that are made are going to have major effects on the choices that are made.*

(Stakeholder; Reintegration; SR#21)

When asked about resources that are needed to mitigate the effects of paternal incarceration and food insecurity, the fathers often discussed how their return to community was impacted by vulnerabilities such as age, illness, and social isolation and that meaningful rehabilitation was critical.

*..to foster rehabilitation..okay..It has to be within different categories. There is the hard core criminal. There’s the medium guy..For me, for example, I am rehabilitating myself. I am part of the community, working hard, providing a service that is important, hiring people and so on. This because..I have the ability, knowledge and education and so on – to say okay I have a dollar and make two dollarsv—then four dollars—I am smart enough to do this. When it comes to other people..I don’t believe there is enough support—financially—in order for that person to be establishing themselves..If there is no meaningful rehabilitation you don’t give the tools to rehabilitate.*

(F#5)

Stakeholders tended to emphasize that the impacts of incarceration-related food insecurity also burdens communities, particularly those which had high crime and incarceration rates. As echoed by others [76], conditions such as family connection, residence, income, and basic food access provide the conditions to fully develop relationships with one’s community. Others spoke about the need for personalized approaches, collaborative action from diverse stakeholders, and integrating participatory strategies.

*…there has to be a space where a person has a capacity to explore parts of themselves that are more in that belief and value level rather than just behaviour..it’s difficult to in my opinion to do this without doing a personal plan for every person to say in light of what your experience is, in light of the culture your part of, in light of your background and the trauma you’ve experienced and in light of your addiction, let’s have a conversation that tailors a plan to you as an individual...*

(SR#19)

*It needs to be people working together—not just the folks working in prison and with community programs. There needs to be a way of figuring out how a family is being affected by the dad being in prison and then coming up with solutions to make things better..especially for the kids.. any ideas for improving the situation needs to include those affected by the problem…*

(WFI#4)

### 3.3. Conceptual Framework

**Figure 1:**
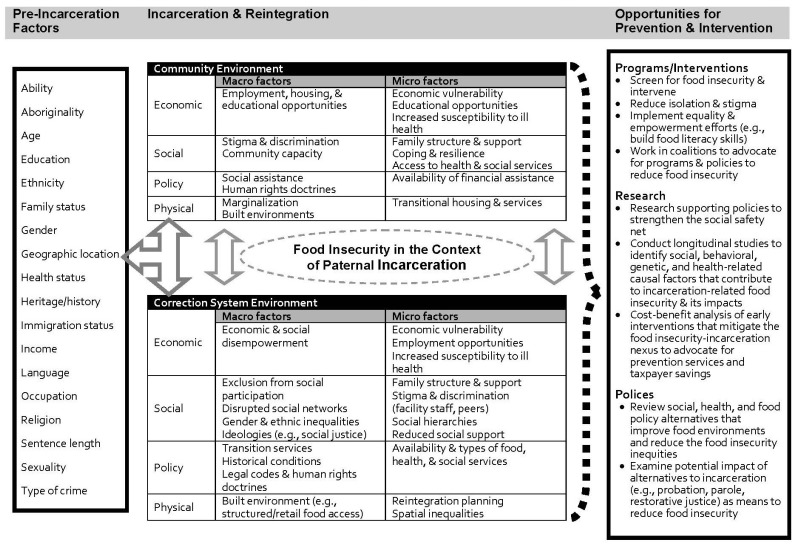
Factors influencing family food insecurity related to paternal incarceration.

The thematic analysis of textual data from those experiencing food insecurity and the stakeholders that work with these individuals helped to shape a conceptual framework (Figure 1) that illustrates the dynamic interplay among micro-, meso- and macro-level factors that contribute to incarceration-related family food insecurity [77,78,79,80]. As depicted in the framework, food insecurity in the context of paternal incarceration results from continuous interactions between the present contexts and processes combined with individual, systemic, and historical factors [81]. For example, inter-related components of employment, income, education, race, gender experience, housing, and geography may intersect with systemic barriers (e.g., discrimination, limiting policies) via multiple socio-ecological influences that forces individuals to the margins of society, entrenches their food insecurity, and perpetuates inter-generational inequalities.

The framework also helps to illustrate and explain how adversity resulting from paternal incarceration (micro-level factor) and community level factors (meso-level) such as proximity to family, cohesion, and disorganization influence family food insecurity [19,82,83]. Societal reintegration then becomes challenged and the access to and effective use of public and non-profit social services becomes critical [49]. It is acknowledged that programs and policies currently have an important role in the lives of families impacted by incarceration-related food insecurity including the provision of community supervision, health care, public transportation, and social services. Thus, policymakers and the resources they have access to, can be particularly influential in implementing interventions that foster the well-being of families impacted by incarceration-related food insecurity. Within the section of the framework “Opportunities for Prevention and Intervention”, various programmatic, investigative, and policy considerations that could intercept the effects of family food insecurity are outlined. The framework integrates existing literature with the findings of our study to suggest interventions to mitigate incarceration-related food insecurity.

## 4. Discussion

The primary goal of this study was to better understand the factors and consequences related to family food insecurity in the context of paternal incarceration. Using interpretative thematic analysis that applied family impact and intersectional theories, it was found that the stressors of family food insecurity and paternal incarceration coexist and mutually reinforce one another. The added burden of food insecurity when fathers were incarcerated further eroded the well-being of family members, which, subsequently, exacerbated the effects of food insecurity. At broader levels, stigmas and cultural norms that emphasize self-sufficiency contributed to the cyclical relationships that exist between paternal incarceration and food insecurity. Food insecurity, as a collateral consequence of incarceration, interacts with different economic, social, policy, and physical circumstances that converge on families and deepens their marginalization. The following discussion highlights potential mechanisms in which paternal incarceration compromises family food security and identifies components of the framework that can be leveraged as strategies to move forward.

### 4.1. Potential Mechanisms

Consequences of food insecurity related to paternal incarceration occur for both the offender and their family members. Fathers living in a correctional facility experience food insufficiency [84] as a result of changes in the types, frequency, and variety of foods consumed. In addition, incarcerated fathers are excluded from the labor market which deprives their families of a source of income [3,85] and increases their vulnerability to food insecurity. Paternal incarceration also threatens the earning power of family members, who may sacrifice work time to perform tasks previously done by the father [86] or struggle to cover incarceration related expenses (e.g., legal representation, costs of maintaining contact through visits) [13]. A family’s financial instability can persist beyond the period of incarceration as ex-prisoners face labor market challenges during reintegration [16,17]. During incarceration, parental relationships tend to dissolve [87] which can increase the likelihood of food insecurity as earnings may no longer be shared [3]. These economic hardships [4] may elevate the family’s stress levels [21] and impede their ability to manage family food resources.

Beyond financial influences are other disruptive factors such as chronic disease, family stresses, and poor function that increase risk for incarceration-related food insecurity [62]. As described by others [61], the lived experience of family food insecurity and paternal incarceration contributes to unfavorable outcomes such as household instability, impairments in mental and physical health, social exclusion, and behavioral challenges in children [5,21,88,89,90]. These effects are perpetuated by structural constraints such as Canada’s current social safety net which provides limited financial supports and policies that govern program assignments and payments for incarcerated individuals. Moreover, stigmas associated with both incarceration and food insecurity erode familial relationships and create motivational barriers to participate in society.

### 4.2. Implications for Practice

This study’s results and proposed framework (Figure 1) shows that the conditions that lead to food insecurity in the context of paternal incarceration are dynamic, and that individual experiences depend on circumstances, history, and opportunities [91,92]. For example, poverty, as a determinant of health, interacts with and reinforces other determinants which then contribute to increased costs in health, social, and justice systems. To address food insecurity in the context of paternal incarceration, downstream and upstream reforms are needed such as screening for food insecurity, creating equities in income, social support, and health, and, where feasible, exploring incarceration and reintegration alternatives. Currently strategies to alleviate food insecurity in Canada include food-based (e.g., charitable programs, community-based interventions) and income responses. Of these two options, income-based responses such as policies that promote income security through employment policies, income transfers, tax exemptions and credits, are more effective at reducing food insecurity [5]. However, in the context of paternal incarceration, approaches to mitigate food insecurity must consider factors such as family justice and developing effective re-entry programs such as supporting access to appropriate living arrangements. Although current health, social, and justice systems are resource-constrained, continued neglect in addressing food insecurity will only perpetuate the human and economic costs that stem from this inequity.

The proposed framework (Figure 1) highlights the need to focus on intersecting societal, policy, community, family, and individual factors to address family food insecurity in the context of paternal incarceration. Historically, the focus of most policies and research related to incarceration has not considered the socio-political contexts that may impact food insecurity. Attention must be directed to the influence of macro-level factors (e.g., housing policies), how incarceration intensifies inequities such as food insecurity, and long-term solutions that takes into account the multidimensional nature of family food insecurity. Examples of a re-entry approaches that would be beneficial for alleviating food insecurity include training farms that build food literacy and skills of incarcerated individuals. These programs foster food connections and improve employment potential of incarcerated individuals [93]. In some instances, these farms or gardens are located within communities and are sources of food provision for residents. These types of approaches align with goals of meaningful rehabilitation and restorative justice [94].

### 4.3. Limitations

Our results may be limited due to selection bias. We primarily recruited individuals who were associated with the agencies of our community partners and therefore may not have reached those experiencing extreme food insecurity. In our sampling, we did recruit people who had at some point encountered issues with access to food. In our experiences with the participants this meant many things such as having issues with the quality of the food offered in facilities to having the need to access emergency food relief services during reintegration. Participants that were interviewed had been exposed to varying levels of hardships and food insecurity. Secondly, part of our sample were stakeholders that were both directly and indirectly involved with corrections and societal reintegration services who could speak about the experiences of different individuals. Finally, we hired peer researchers who had experienced successful societal reintegration and overcame food insecurity issues to provide further insights. By integrating multiple perspectives we believe that the described mechanisms generalize to the scope of the study.

The interviews were done with fathers who were formerly incarcerated and may not necessarily reflect current experiences with CSC services. For example, based on field observations and stakeholder interviews, current programs that build food skills and changes in some CSC food delivery systems were highlighted. The interview data from fathers and their partners were from those living in British Columbia and thus may not reflect the experiences of those residing in other Canadian provinces or countries. However, many of the respondents had been in CSC facilities across Canada and provided similar accounts related to experiences of food insecurity. Furthermore, some stakeholder interviews were with individuals residing out of province which helped strengthened the study’s representativeness. The purpose of the proposed framework is to help foster understanding about how food insecurity manifests in the context of paternal incarceration, however, a framework that includes specific evidence about food insecurity interventions in the context of incarceration in relation to health, social and justice outcomes, would provide more guidance on program and policy development.

## 5. Conclusions

The use of ethnographic, family impact, and intersectional-based analytic approaches underscored the importance of understanding the multidimensional nature of food insecurity in the context of paternal incarceration. Furthermore, it clarified the need for interventions that reflect factors such as age, gender, socio-economic status, ethnicity/race, and marital status that shape food insecurity in the context of paternal incarceration. There are many opportunities to guide best practices that would foster supportive food environments for families affected by paternal incarceration and help reduce associated health, social, and justice system costs. Collaborative action among diverse stakeholders—practitioners, policy makers, health, social, and criminal justice-based agencies, researchers—that include participatory approaches could facilitate the change necessary to reduce inequities associated with paternal incarceration and food insecurity. In particular, interventions aimed at prescriptive ethics, social justice, health promotion, and meaningful rehabilitation show promise at mitigating the familial and intergenerational effects of paternal incarceration and food insecurity.

## Figures and Tables

**Table 1:** Description of Participants.

Description	# of People
**In Depth Individual Interviews**	
Stakeholders ^a^:	29
Corrections system including correctional officers, parole officers, administrators, health practitioners (*n* = 16)	
Reintegration services ^a^ (*n* = 13)	
Formerly incarcerated fathers (10 lived with their children prior to incarceration)	11
Partners of formerly incarcerated fathers (with children)	7
**In Depth Focus Group Interviews**	
Three focus groups that included:	16
Current/ former incarcerated fathers; one lived with their children prior to incarceration (*n* = 2)	
Partners of currently or formerly incarcerated fathers (*n* = 3)	
Stakeholders ^a^ (*n* = 11):	
-Within the corrections system (*n* = 3)	
-Involved in societal reintegration (*n* = 8)	

^a^ Examples: L.I.N.C, Elizabeth Fry Society, The John Howard Society of Canada, St. Leonard’s Society, Correctional Service Canada, Hope Central, Salvation Army, Lookout Society.

## Supplementary Materials

The following are available online at https://www.mdpi.com/1660-4601/16/5/776/s1, S1: Individual and Focus Group Interview Guides.
