# Supplementary material for: Food Security in the Context of Paternal Incarceration: Family Impact Perspectives
Source: Int J Environ Res Public Health. 2019 Mar 4;16(5):776. doi: 10.3390/ijerph16050776 (PMC6427226; doi:10.3390/ijerph16050776)
Supplement: Supplementary file 1 [file ijerph-16-00776-s001.pdf]

Individual and Focus Group Interview Guides

Person with Lived Experience of Incarceration

1. Tell me about yourself and why you are interested in participating in this interview?
2. Describe your relationship with food while incarcerated and as you transitioned back into the community.
3. What are factors that affect/affected your relationship with food in this context? How might they be changed to foster a person's health who is incarcerated?
4. How might these factors influence your role as a volunteer, staff member, person living in the community?
5. What do you think is the main purpose of the agency(ies) who assist you?
6. What do you think is the main purpose of providing food and food programming in a correctional facility?
7. What do you think is the biggest problem with accessing food after you leave the correctional facility and transition in the community?
8. Who do you think should be responsible for helping people who are transitioning from a correctional facility to access appropriate food? Explain why.
9. Is there anything else you would like to tell me that we haven't discussed?

Stakeholder

1. Please describe the role(s) you have been involved in that are related to the Canadian correctional system.
2. Tell me about the program/organization(s) that you are connected with and what they offer to people who are currently or formerly incarcerated.
3. Tell me about the people you see who are currently or previously involved with federal corrections system (e.g., services provided).
4. What do you think are the major issues (e.g., social, health) people who are currently or previously involved with the federal corrections system face?
5. Tell me your thoughts about services that are aimed at societal reintegration?
6. What factors do you think help with successful societal reintegration?
7. What sort of factors help or hinder those who are currently or previously involved with the federal corrections system when it comes to accessing food? Services related to food and nutrition?
8. What type of resources do you think would help people who are transitioning from incarceration to access appropriate food? To support good nutrition and healthy eating?
9. Is there anything else you would like to tell me that we haven't discussed?
